# A survey of nurses' awareness of infection control measures in Baranya County, Hungary

**DOI:** 10.1002/nop2.897

**Published:** 2021-05-05

**Authors:** Sahar Hammoud, Haitham Khatatbeh, Afshin Zand, Béla Kocsis

**Affiliations:** ^1^ Doctoral School of Health Sciences Faculty of Health Sciences University of Pécs Pécs Hungary; ^2^ Department of Medical Microbiology and Immunology Medical School University of Pécs Pécs Hungary

**Keywords:** awareness, Hungary, infection control, knowledge, nurse

## Abstract

**Aim:**

This study aimed to assess nurses' awareness of infection control (IC) measures in Baranya County, Hungary.

**Design:**

A cross‐sectional survey.

**Methods:**

The study used the infection control standardized questionnaire to assess nurses' awareness in standard precautions (SP), healthcare‐associated infections (HAIs) and hand hygiene (HH). Data collection was done from two hospitals in February and March 2020. SPSS was used for statistical analysis.

**Results:**

The study included 121 nurses. The mean scores were 16.55 ± 2.69 for IC overall awareness, 10.10 ± 1.58 for SP, 2.07 ± 0.71 for HAIs and 4.38 ± 1.47 for HH. Acceptable scores were reached in overall awareness and SP. The overall and HAIs' scores significantly differed across educational degrees. The difference in SP mean ranks was statistically significant across hospital types. The low HAIs and HH scores highlight the need to enhance IC trainings in Hungarian hospitals and improve nurses' knowledge on IC.

## 1 INTRODUCTION

Healthcare workers (HCWs) employed in hospital settings regularly deliver healthcare services to patients with unknown status of blood‐borne diseases, such as Hepatitis B, Hepatitis C and human immunodeficiency virus (HIV) (Regina et al., 2002; Porto & Marziale, 2016) in addition to dealing with the increasing incidence and emergence of infectious diseases worldwide (Colet et al., 2017). This exposure places HCWs at risk of acquiring occupation‐related viral infections. The literature has shown that nurses have the highest risks of acquiring occupation‐related infections among HCWs since they have the most frequent direct interactions with patients while providing care (Porto & Marziale, 2016). According to the Centre for Disease Control and Prevention (CDC), nurses are the most HCWs whom are repeatedly included in either documented or possible occupationally acquired HIV infections (Regina et al., 2002).

## 2 BACKGROUND

As per the World Health Organi​zation (WHO), hundreds of millions of patients acquire healthcare‐associated infections (HAIs) annually, where seven patients in developed and 10 in developing countries acquire a minimum of one HAI per 100 hospitalized patients at any given time (World Health Organization, 2015). Since decades till present, the CDC has firmly recommended infection control (IC) precautions to prevent the transmission of infections, disease outbreaks and assure the safety of HCWs (Wu et al., 2008). Despite the development and improvement in the IC programmes in hospitals, low compliance with IC practices has been reported among HCWs worldwide over the years (Valim et al., 2013). This low compliance remains to be linked with HAIs (Colet et al., 2017). A main contributing factor to the low compliance is lack of awareness (Sodhi et al., 2013). Having the most frequent direct interactions with patients while providing daily care, nurses have the highest risks of transmitting HAIs among patients and HCWs (Alrubaiee et al., 2017; Colet et al., 2017; Cruz & Bashtawi, 2016; Cruz et al., 2015).

In addition to education and training, the CDC recommends periodic assessment of HCWs' knowledge and compliance with IC practices to control and prevent the transmission of HAIs (AL‐Rawajfah, & Tubaishat, 2017). The CDC emphasizes that education on the principles and practices for preventing the transmission of infections shall be given to all HCWs. These education and training programmes should be conducted regularly. However, new updates of IC guidelines are to be added to upcoming trainings (Center for Disease Control & Prevention, 2007).

Many international studies have examined the level of nurses' knowledge and awareness of IC practices. In Europe, similar studies were conducted that included HCWs, nurses and nursing students. Tavolacci et al. (2008) surveyed 350 healthcare students (medical, nursing, assistant radiologists and physiotherapists) using the infection control standardized questionnaire (ICSQ) in France, to assess their knowledge of IC and their source of information. The mean overall IC knowledge score was acceptable (21.5 of 30). However, the knowledge scores differed across specific IC areas. The highest knowledge scores were among standard precautions (SP) and hand hygiene (HH), and the lowest was among HAIs.

In another study, (D'Alessandro et al., 2013) surveyed 1,461 nursing and medical students using the ICSQ in Italy, to determine their knowledge regarding the prevention of HAIs. The mean overall IC knowledge score was acceptable (18.1 of 25). However, the knowledge scores varied across different IC areas. The acceptable knowledge score was achieved in SPs only, whereas HH and HAIs' scores were not acceptable.

(Butsashvili et al., 2010) conducted a study in Georgia using a developed questionnaire where 433 nurses and physicians were surveyed to determine their level of knowledge in SP and HAIs and their source of information. 41.1% of the participants had an acceptable overall knowledge score (a score of ≥6 over 10).

Although the relevant research specific to nurses solely is limited in Europe, these studies highlight the need for more nursing education and training in IC. Moreover, up to our knowledge, no such studies exist in Hungary. So, we aim in this study to assess the level of nurses' awareness with IC measures in Hungary, more specifically in Baranya County.

## 3 METHODS

### 3.1 Study design, setting and sample

This study is part of a broad research about the impact of nurses' awareness of IC measures on patient and family education among Hungarian hospitals. The study used a cross‐sectional design. In the present study, a convenience sample of two hospitals in Baranya County was selected. The first was a university hospital and the second a city hospital.

In the current study, our sample consisted of 121 nurses, with a significance level of 0.05, confidence interval 0.95, effect size 0.317 and ability of representing the population of 0.96 as a result of the post hoc power analysis that was done using (G*Power, 2020). This result means that our sample size is enough to make statistical conclusions.

All nurses working in the inpatient units were eligible for the study. Consent forms including information sheets and self‐administered questionnaires were distributed to all eligible nurses who were present at the hospitals and willing to participate. The questionnaires and consent forms were distributed by the nurse managers as printed copies. The papers were left with the nurses who gave them back to the nurse manager when completed. The researchers collected the completed questionnaires 2 months later. Data collection was started in early February 2020 and was finished by the end of March 2020. In total, 123 of 180 nurses in the two hospitals completed the questionnaires, yielding a 68.3% response rate.

It should be noted that having an IC programme is an obligation not only in Hungary but in any European Union (EU) member states. As per the (European Commission, 2009) guidelines, the IC programme aims to reduce the risks of HAIs in healthcare institutions through the development and implementation of all necessary IC measures. All healthcare institutions operating in the EU should define responsibilities at all organizational levels, organize support, allocate resources and monitor performance through setting up evaluation procedures. Healthcare‐associated infections surveillance system should also exist at the institutional and national level. In addition, to educating and training HCWs regularly on IC measures and promoting patient safety through reducing adverse events occurrence. Similarly, the Hungarian accreditation standards for inpatient and outpatient organizations and public pharmacies were prepared with a prominence on patient safety ensuing the requirements of the International Society for Quality in Healthcare (Dombrádi et al., 2018). These standards include specific IC standards. Furthermore, there are guidelines that have been established by the Hungarian Ministry of Health, and governmental regulations that determine IC practices among healthcare institutions in Hungary (Weinshel et al., 2015).

### 3.2 Data collection

The study used a modified version of the ICSQ adopted from a French study (Tavolacci et al., 2008). The approval for using the questionnaire was taken from the Cambridge University Press. The questionnaire included two parts, the demographics part to collect information about gender, age, hospital type, nursing unit, educational degrees, years of service and training attended in IC. The second part involved 23 closed‐ended questions (instead of 25) to assess the awareness of nurses in three different IC areas, HAIs (three questions, question 1A, 1B and 1C), HH (eight questions, question 3A, 3B, 3C and 3D, and question 6A, 6B, 6C and 6D) and SP (12 questions, question 2A, 2B, 2C and 2D, question 4A, 4B, 4C and 4D, and question 5A, 5B, 5C and 5D). Two questions from HAIs' area were cancelled (prevalence of HAIs in Hungary and the number of annual death due to HAIs) to adapt to the Hungarian situation. Response of each question was coded and scored as aware (1) or not aware (0). A maximum score of 23 was obtainable, the cut‐off for an acceptable or high awareness level of each area (HAIs, HH and SP) and the overall awareness was 70%, as per the study by (Tavolacci et al., 2008). This was equivalent to a score ≥2.1 for HAIs, ≥5.6 for HH, ≥8.4 for SP and ≥16.1 for the total awareness. So, a score less than the mentioned values was considered as a non‐acceptable or of low awareness level. According to the translation and validation guidelines of (Sousa & Rojjanasrirat, 2011), the questionnaire was translated first from English (the original language of the questionnaire) into Hungarian by two different individuals. Then, the two translated versions were compared by a committee approach; so, a unified Hungarian version resulted. After that, two blind back translations into English were done for the resulted Hungarian version by two individuals. Then, a two‐step comparing process was done. First, a comparison between the two back‐translated versions of the questionnaire, and second, a comparison of the two back‐translated versions with the original English version of the questionnaire to assess similarities of the questionnaire items, writings, meanings and significance (the comparison was done by a Hungarian linguistic associate professor, who is knowledgeable about health terminology, and a bilingual physician who is well knowledgeable about IC). At the end of this step, a final Hungarian version of the questionnaire resulted. A pilot study on 15 nurses yielded a Cronbach's alpha of 0.76 compared to 0.61 of the original questionnaire (Tavolacci et al., 2008) which shows a good reliability coefficient.

### 3.3 Data analysis

Statistical Package for the Social Sciences (SPSS) version 20 was used for data management and analysis. Descriptive statistics were produced in terms of frequencies and percentages for categorical variables, with means and standard deviations (*SD*) for continuous variables. Kolmogorov–Smirnov test was used to evaluate the normal distribution. The non‐parametric Mann–Whitney *U* test and Kruskal–Wallis test were used to compare the difference in awareness mean ranks across independent variables. The significance level was set at *p* < .05. In order to manage missing data, incomplete questionnaires were discarded.

### 3.4 Ethical considerations

Ethical approval was granted by the Regional Research Ethics Committee of the Medical Centre, Pécs, Hungary (Record number: 7862 – PTE 2019).

## 4 RESULTS

### 4.1 Demographic characteristics

From the 123 returned questionnaires, two were discarded due to incomplete data so, the final included papers were 121. Of these 121, 93.4% were females. Ages ranged from 20–63 years, with a mean age (± *SD*) of 39.64 ± 11.32 years. Out of all nurses, 74.4% were working in a university hospital. Participants' detailed demographics are presented in Table 1.

**TABLE 1:** Demographic characteristics of study participants (*N* = 121)

Demographics	Number of respondents *n* (%)
Gender	
Female	113 (93.4%)
Male	8 (6.6%)
Hospital type	
University	90 (74.4%)
City	31 (25.6%)
Department	
Medicine	24 (19.8%)
Infectious	7 (5.8%)
Surgery	41 (33.9%)
ICU	13 (10.7%)
CICU	4 (3.3%)
NICU	1 (0.8%)
OB‐GYN	9 (7.4%)
Haematology	2 (1.7%)
Oncology	3 (2.5%)
Paediatrics	8 (6.6%)
PICU	9 (7.4%)
Educational degrees	
University	23 (19%)
Secondary school with nursing specification	56 (46.3%)
Lower nursing degrees	42 (34.7%)
Years of service at the hospital	
<1 year	8 (6.6%)
[1–5 years]	27 (22.3%)
[6–10 years]	14 (11.6%)
>10 years	72 (59.5%)

Abbreviations: CICU, cardiac intensive care unit; ICU, intensive care unit; NICU, neonatal intensive care unit; OB‐GYN, obstetrics‐gynaecology; PICU, paediatric intensive care unit.

Almost all nurses (99.2%) attended trainings on IC topics at their current hospital. 71.9% attended the training a year ago. Hand hygiene was the most topic nurses trained on (95.9%), while managing hospital blood/body fluid spills was the least (52.9%). The numbers and percentages of training attended on each topic are shown in Table 2.

**TABLE 2:** Infection control training topics attended by nurses at their hospitals (*N* = 121)

Infection control training topics	Number of respondents *n* (%)
Hand Hygiene	116 (95.9%)
Usage of personal protective equipment	97 (80.2%)
Healthcare‐associated infections	83 (68.6%)
Standard precautions	74 (61.2%)
Isolation precautions	67 (55.4%)
Managing hospital blood/body fluid spills	64 (52.9%)

### 4.2 IC awareness

The 23 questions of the awareness part with the numbers and percentages of correct answers are shown in Table 3.

**TABLE 3:** Infection control awareness questions of respondents who gave correct answers (*N* = 121)

Question	Correct answer	Respondents with correct answers *n* (%)
1. Healthcare‐associated infections		
A. The environment (air, water, inert surfaces) is the major source of bacteria responsible for nosocomial infection	False	64 (52.9%)
B. Advanced age or very young age increases the risk of nosocomial infection	True	68 (56.2%)
C. Invasive procedures increase the risk of nosocomial infection	True	119 (98.3%)
2. Standard precautions		
A. Include the recommendations to protect only the patients	False	120 (99.2%)
B. Include the recommendations to protect the patients and the healthcare workers	True	119 (98.3%)
C. Apply for all patients	True	92 (76%)
D. Apply for only healthcare workers who have contact with body fluids	False	109 (90.1%)
3. When is hand hygiene recommended?		
A. Before or after a contact with (care of) a patient	False	89 (73.6%)
B. Before and after a contact with (care of) a patient	True	121 (100%)
C. Between patient contacts	True	99 (81.8%)
D. After the removal of gloves	True	97 (80.2%)
4. The standard precautions recommend use of gloves		
A. For each procedure	False	26 (21.5%)
B. When there is a risk of contact with the blood or body fluid	True	108 (89.3%)
C. When there is a risk of a cut	True	90 (74.4%)
D. When healthcare workers have a cutaneous lesion	True	108 (89.3%)
5. When there is a risk of splashes or spray of blood and body fluids, the healthcare workers must wear		
A. Only mask	False	111 (91.7%)
B. Only eye protection	False	116 (95.9%)
C. Only a gown	False	112 (92.6%)
D. Mask, goggles and gowns	True	111 (91.7%)
6. What are the indications for the use of alcohol‐based hand rub (on unsoiled hands)?		
A. Instead of a traditional handwashing (30 s)	True	50 (41.3%)
B. Instead of an antiseptic handwashing (30 s)	True	34 (28.1%)
C. Instead of surgical handwashing (3 min)	True	9 (7.4%)
D. A traditional handwashing must be done before handwashing with alcohol‐based hand rub	False	31 (25.6%)

The mean overall awareness score (± *SD*) was 16.55 ± 2.69. The mean score (± *SD*) was 10.10 ± 1.58 for SP, 2.07 ± 0.71 for HAIs and 4.38 ± 1.47 for HH. 61.2% of nurses had a high total awareness level, 81.8% had a high awareness in SP, 28.1% had a high awareness in HAIs and 21.5% had a high awareness in HH.

The overall awareness score did not significantly differ across gender, hospital type, age, departments and years of service. However, it significantly differed across different educational degrees (*χ* ^2^(2) = 8.225, *p* = .016), with a mean awareness rank of 75.52 for university degrees, 63.04 for secondary school with nursing major and 50.32 for other lower nursing degrees. Furthermore, there was a statistically significant difference in the HAIs score among different educational degrees (*χ* ^2^(2) = 6.604, *p* = .037) with a mean awareness rank of 74.98 for university degrees, 60.74 for secondary school with nursing major and 53.69 for other lower nursing degrees. Finally, the difference in SP mean ranks was statistically significant across different hospital types (64.88 for university hospital, 49.74 for city hospital, *U* = 1,046, *p* = .03).

## 5 DISCUSSION

The current study assesses the nurses' awareness level of IC measures in Baranya County, Hungary. The participants demonstrated an acceptable overall mean awareness score (16.55 ± 2.69 from a total of 23). These results are consistent with the European studies by (D'Alessandro et al., 2013; Tavolacci et al., 2008). However, participants' awareness differed across the three IC areas, where the acceptable awareness score was only achieved in SP, which is similar to the study by (D'Alessandro et al., 2013). The mean score of HAIs was low but, was better than that by (D'Alessandro et al., 2013; Tavolacci et al., 2008), while the worst score was for HH which was lower than the scores by (D'Alessandro et al., 2013; Tavolacci et al., 2008).

The highest awareness score was achieved for SP. Standard precautions are considered the chief strategy to prevent the transmission of occupation‐related infections among HCWs and patients (Center for Disease Control & Prevention, 2007; Duarte Valim et al., 2016). However, our results could not be considered promising since acceptable scores were not reached in both HH and HAIs areas. On the other hand, almost all nurses attended training on IC topics at their current hospital, which is similar to the results of (Hammoud et al., 2017). 71.9% of nurses attended the training a year ago, which indicates that hospitals are implementing IC training periodically as per the CDC recommendations (Center for Disease Control & Prevention, 2007). In addition, the highest training score was in HH (95.9%) but, the lowest mean awareness score among the three IC areas was in HH as well. Moreover, the study shows that nurses had insufficient knowledge about the indications of the use of alcohol‐based hand rub on unsoiled hands instead of traditional handwashing. So, the knowledge on HH should be improved since, it is the most effective measure to reduce the spread of infections inside hospitals (Hammoud et al., 2020; Kelcíkova et al., 2012; Mathur, 2011; Salama et al., 2013) and is one of the main recommendations of the CDC and the WHO to fight against coronavirus diseases (COVID‐19) pandemic that the whole world is facing nowadays (Center for Disease Control & Prevention, 2020a, 2020b; World Health Organization, 2020). At the same time, trainings on other IC topics should also be enhanced, for example; managing hospital blood/body fluid spills and isolation precautions which were the least attended by nurses because of their importance in protecting the HCWs from occupational infectious agents.

Concerning HAIs awareness, the results show that nurses were not aware that the environment is not the major source of bacteria responsible for HAIs and that advanced age or very young age increases the risk of HAIs. Additionally, only 68% of nurses mentioned that they were trained on HAIs. This highlights the need to emphasize the training on HAIs as a way to increase knowledge and awareness.

The mean overall awareness score and HAIs score differed significantly among different educational degrees, where nurses with a university degree had a higher mean than those with lower degrees, and this could be explained due to the fact that the curricular content of undergraduates does not stress on IC practices the same way that the university curriculum does (Amin & Al Wehedy, 2009; El‐Gilany et al., 2012).

This study sheds light on the IC areas where deficiencies in awareness were found among Hungarian nurses. Hospitals are encouraged to put more efforts into their IC training programmes and increase nurses' knowledge on IC measures since, they have a major role in preventing the transmission of HAIs thus, decreasing patients' length of stay, and providing staff safety. This could be done by applying periodic competencies to assess IC knowledge deficiencies among nurses, prioritize needs and act accordingly. Further studies are recommended to include a larger sample size, nurses from inpatients and outpatients units, and more diverse geographical settings.

## 6 LIMITATIONS

Our study has some limitations; first, the study included two hospitals from only one County in Hungary. So, the results may not be generalized and do not necessarily reflect the situation in all Hungarian hospitals. Nevertheless, we believe that this study could increase the attention towards IC programmes in Hungarian hospitals and encourage similar studies at the national level. Second, our inclusion of only inpatient units and using a convenience sampling may have resulted in selection bias. Third, having a relatively small sample size may have overestimated the awareness score level. Hence, further studies are recommended to include a larger sample size.

## 7 CONCLUSION

In conclusion, this study reveals an acceptable (a score ≥70%) IC overall awareness score (16.55 ± 2.69) among nurses in Baranya County, Hungary. The awareness scores varied across IC areas; the score of SP was acceptable (10.10 ± 1.58), while the scores of HAIs (2.07 ± 0.71) and HH (4.38 ± 1.47) were not. Hospitals are encouraged to enhance their IC training programmes and increase the nurses' knowledge on IC measures. Further studies are needed to assess IC awareness of nurses at the national level.

## CONFLICT OF INTEREST

The authors declare no conflict of interest.

## AUTHOR CONTRIBUTIONS

Sahar Hammoud and Béla Kocsis: Conceptualization and the design of the study. Sahar Hammoud and Afshin Zand: Acquisition of data. Sahar Hammoud and Haitham Khatatbeh: Analysis and interpretation of data. Sahar Hammoud: Drafting the paper. All authors revised the paper critically and approved the final manuscript.

## Data Availability

The raw data that support the results of this research are available from the corresponding author upon a reasonable request.
